# The Novel Flax Cultivar Silesia Shows High Morphogenetic Capacity in Tissue Cultures

**DOI:** 10.3390/ijms26188847

**Published:** 2025-09-11

**Authors:** Mateusz Lipiński, Kinga Pilarska-Dudziak, Tadeusz Czuj, Anna Prescha, Wojciech Łaba, Katarzyna Skórkowska-Telichowska, Magdalena Wróbel-Kwiatkowska

**Affiliations:** 1 Department of Biotechnology and Food Microbiology, Faculty of Biotechnology and Food Science, Wrocław University of Environmental and Life Sciences, ul. Chełmońskiego 37, 51-630 Wrocław, Poland; 121224@student.upwr.edu.pl (M.L.); kinga.pilarska-dudziak@upwr.edu.pl (K.P.-D.); wojciech.laba@upwr.edu.pl (W.Ł.); 2 Department of Genetics, Plant Breeding and Seed Production, Faculty of Life Sciences and Technology, Wroclaw University of Environmental and Life Sciences, pl. Grunwaldzki 24 A, 50-363 Wrocław, Poland; tadeusz.czuj@upwr.edu.pl; 3 Department of Dietetics and Bromatology, Faculty of Pharmacy, Wroclaw Medical University, ul. Borowska 211, 50-556 Wroclaw, Poland; anna.prescha@umw.edu.pl; 4 Medical Faculty, Wrocław University of Science and Technology, pl. Grunwaldzki 11, 51-377 Wrocław, Poland; katarzyna.skorkowska-telichowska@pwr.edu.pl; 5 Department of Endocrinology, Jerzy Gromkowski Regional Specialist Hospital, ul. Koszarowa 5, 51-149 Wrocław, Poland

**Keywords:** *Linum usitatissimum* L., callus, regeneration, morphogenetic response, fatty acids

## Abstract

The aim of the present study was to evaluate the morphogenetic efficiency of the new flax (*Linum usitatissimum* L.) cultivar Silesia. The plant material consisted of five ecotypes of Silesia seeds selected by determining the fatty acid composition in individual plants. Thus, five ecotypes with different fatty acid compositions were applied for germination, and the resulting seedlings were used for callus induction. The observed in vitro morphogenic response of explants to the used callus induction medium was 100%, followed by varying development of shoots, with the highest value being 17 shoots per callus, with an average length of 7.15 cm (for ecotype 1). The biochemical analyses showed photosynthetic pigments were significantly affected by the tested ecotypes. The main conclusion is that the selection of plant material as a source of tissue cultures is crucial for efficient regeneration and organogenesis. One factor potentially influencing these processes is fatty acid composition and the ratio of linoleic to α-linolenic acid. A ratio ranging from 1.36 to 1.68 in the source material (seeds) used for initiation of tissue cultures resulted in the highest efficiency of shoot regeneration and number of obtained shoots per callus. A strong negative correlation (−0.78) was observed between shoot regeneration efficiency and the ratio of linoleic to α-linolenic acid in seeds from which the explants were obtained. Moreover, an efficient protocol of micropropagation from callus tissue was established for the new flax cultivar Silesia. An interesting insight into the metabolism of the obtained regenerants allowed us to determine the relationship between the content of chlorophyll and rhizogenesis efficiency. For these two parameters, the calculated correlation coefficient was 0.66. A strong relationship (high correlation coefficient: 0.79) was also established between flavonoid content and length of obtained regenerants, pointing to the developmental role of flavonoids.

## 1. Introduction

The plant material in the present study was flax (*Linum usitatissimum* L.), an annual, self-pollinating crop plant with economical value. The plant is called “golden” [1] because it has high potential for medicine, as well as for industry [2]. What is more, flax is a very useful plant in tissue cultures, and it can be applied for genetic transformation and suspension cultures [3].

Plant regeneration remains a crucial process for plant transformation and propagation of endangered plants, e.g., *Crocus* sp. [4] and *Sarracenia* sp. [5]. Many factors influence this process, including plant genotype and age, the regenerative capacity of cells, conditions of plant growth, and compositions of media used for all transformation steps. The key process for plant transformation is regeneration, during which new plants are developed from explants [6]. This process is also crucial for clonal micropropagation of plants [7]. There are mainly three types of regeneration: somatic embryogenesis, de novo organogenesis, and regeneration of plant tissues [8]. During regeneration, differentiated plant cells produce a pluripotent cell mass, called a callus, which regenerates in organs (shoots, roots), especially after induction of plant growth regulators [9]. During the regeneration process, cell differentiation is caused by changes in the gene expression, i.e., overexpression of transcription factors involved in morphogenesis [6], and reorganization of chromatin [10], which alters perception of plant growth regulators and induces developmental pathways that enable the transformation from somatic to embryogenic development.

The regenerative capacity of cells can vary depending on the plant genotype, type of cultivar, type of explant, and even the type of cells [10]. It was reported that the type of explant can influence metabolite synthesis, resulting in a callus [4]. Among the many described factors which can influence efficient callus formation, fatty acid profile is notably undervalued. Fatty acids are membrane components but also play a role in the response of plants to stress conditions, i.e., higher levels of fatty acid unsaturation allow plants to survive at low temperatures [11]. However, research has also demonstrated an association between fatty acid composition (unsaturation level) and the rate of cell division [11], and during callus formation, intensive cell division occurs. Thus, the main aim of this study was to evaluate the morphogenetic capacity of the new flax cultivar Silesia, and the second aim was to investigate the relationship between the different amounts of fatty acids in flax seeds of the Silesia cultivar and organogenesis as well as callus formation efficiency.

It should be pointed out that no data in tissue cultures have been presented so far for Silesia cultivar. Although flax is a known industrial and medicinal crop [12,13] cultivated since ancient times [14], the cultivar Silesia is a new Polish oilseed cultivar, registered in 2020, and no transformation or regeneration processes have been performed for it so far. The seeds of the cultivar Silesia possess a medium level of omega 3 fatty acids [15], and this cultivar has been analyzed so far only from a nutritional point of view. Fatty acid analysis of oil obtained from Silesia seeds revealed a unique composition: a lower level of α-linolenic acid C18:3 (29.91%) and a higher level of linoleic acid C18:2 (38.75%) [15]. The determination of the regenerative capacity of each genus and cultivar is crucial to assess its significance for genetic transformation application and obtaining new crops. Thus, plant materials in the present study were flax seeds obtained from the different flax ecotypes (of Silesia cultivar), cultivated in different areas and having a varied fatty acid composition. There are many literature positions describing the importance of omega 6 to omega 3 fatty acid ratio for animals and humans, which follows from the fact that these fatty acids cannot be synthesized by humans and are classified as essential fatty acids [16]. The question is whether this ratio is also important for plant physiology and for plant seeds because their formation is a key phenomenon in plant propagation [17].

## 2. Results and Discussion

### 2.1. Establishment of Germination Efficiency

**Figure 1:**
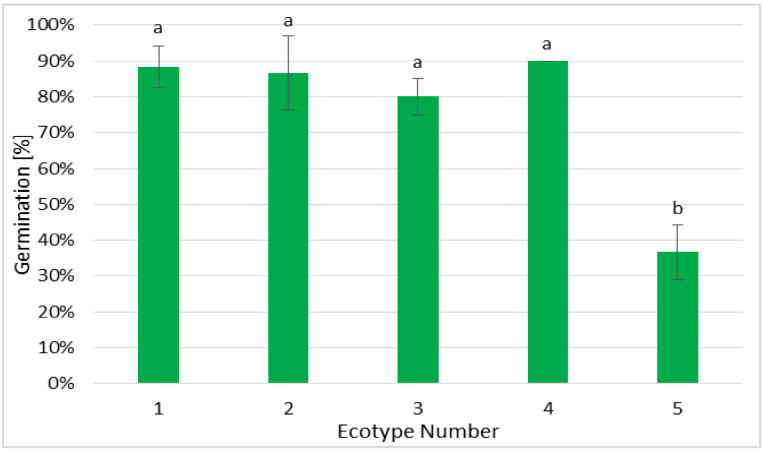
Germination efficiency established for different ecotypes of Silesia seeds. The seeds were germinated in sterile tissue cultures as described in the Section 3. For each ecotype, 60 seeds were used. Values are presented as means ± SD. One-way ANOVA followed by the least-significant-difference (LSD) test was used for statistical analysis. Different letters indicate statistically significant differences at *p* ≤ 0.05.

The germination efficiency of tested ecotypes of Silesia seeds varied depending on the ecotype from 38% to 90% (Figure 1). The lowest germination efficiency was observed for ecotype 5, which exhibited 44.23% of linoleic acid and 26.29% of α-linolenic acid in the seeds (Table 1). The calculated ratio of linoleic (C18:2) to α-linolenic acid (C18:3) was equal to 1.68 (Table 2). The seeds of the other ecotypes exhibited comparable germination efficiency, even though they exhibited different ratios of fatty acids C18:2 to C18:3, e.g., in ecotype 1 the C18:2 to C18:3 ratio was 1.36; in ecotype 2 it was 55.28; in ecotypes 3 and 4 it was, respectively, 0.36 and 6.23. Hence, it may be concluded that the fatty acid composition in seeds was not the only factor impacting the germination efficiency of Silesia seeds. In fact, it was found that lipids (triacylglycerols) from oil bodies in the seeds are degraded during germination and seedling growth and remain a source of carbon until the seedling starts photosynthesis [18]. Thus, the fatty acid composition is crucial for germination and seedling growth, but it has also been reported that not only the lipid composition but also the ability of lipids (fatty acids) to be metabolized into sugars can affect the efficiency of germination and seedling growth [19]. There are many external factors that influence germination efficiency, e.g., water availability, temperature, and internal factors such as fatty acid composition in seeds [20]. However, in the present study, the growth conditions were the same for all tested seeds, so the observed differences may be due to the biochemical composition of seeds.

### 2.2. Frequency of Callus Induction

**Figure 2:**
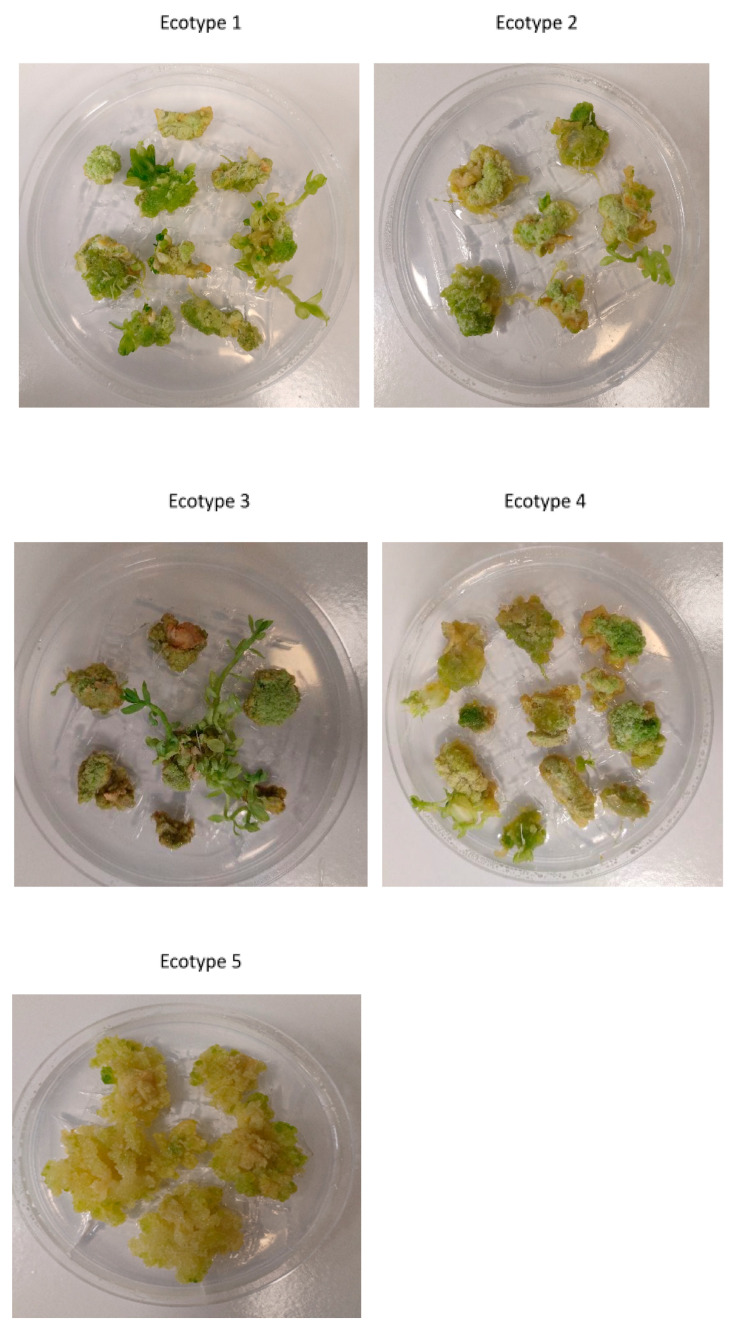
In vitro morphogenic response of different ecotypes of Silesia cultivar to applied callus induction medium (CIM). The photographs show 10-week-old calli, which were subcultured every 2 weeks on fresh CIM medium until the callus was formed. The composition of the CIM medium is described in the Section 3.

The obtained data demonstrated 100% callus induction for all analyzed ecotypes (Figure 2). The calli were green and covered the explants taken for callus induction. The calli from ecotypes 1–4 were non-homogenous with hard fragments, while the calli observed for ecotype 5 were homogenous and friable (Figure 2). In the case of calli from ecotypes 1–3, comparable shoots were visible at the same time, and the rest of them needed more time to form shoots. It can be concluded that all of the used explants (cotyledons) generated a callus, which indicates the very high morphogenetic potential of the tested ecotypes.

It can be concluded that the method used for callus induction (applied composition of CIM medium) was very efficient; used plant growth regulators play a key role in callus induction, cell division, and differentiation [21].

The second reason for the observed phenomenon may be the genotype of tested flax ecotypes, because this is another crucial factor for somatic embryogenesis [22].

For example, it was reported that some fatty acids (VLFAs, very-long-chain fatty acids) may influence the regeneration capacity and the ability of callus formation in *Arabidopsis thaliana* [9,23]. It was suggested that fatty acids (VLFAs) may act as signal molecules that participate in the acquisition of cell competence for callus formation [9]. Very long fatty acids (fatty acids longer than 18C) are synthesized in plants by the elongation of fatty acids and addition of 2C units. In the present paper, VLFAs in flax seeds were not measured, but their precursors (C16 and C18) were observed (Table 1). The performed analyses showed comparable amounts of palmitic acid in all tested cultivars as well as the sum of all C18 fatty acids. This may be the reason for the observed comparable and very high callus formation efficiency.

It was also reported that an *Arabidopsis* double mutant with a reduced NADPH level and decreased amounts of VLFA showed higher callus formation efficiency when compared to wild-type plants [23]. All of these data confirm that fatty acids are crucial for callus formation.

### 2.3. Efficiency of Shoot Regeneration and Rooting

**Figure 3:**
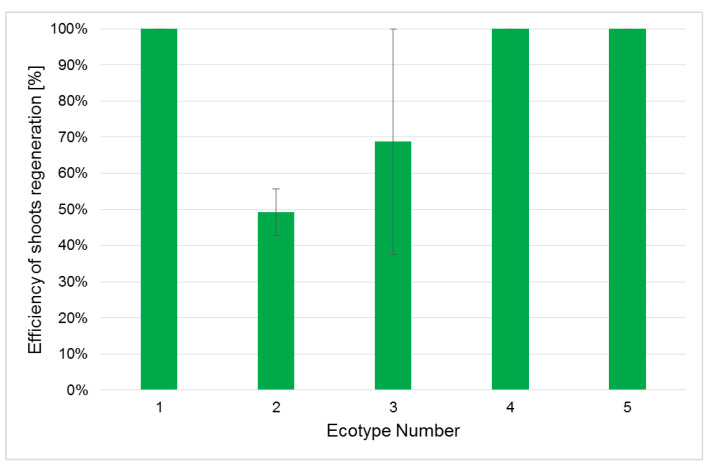
Efficiency of shoot regeneration on explants derived from different ecotypes of Silesia cultivar. The number of generated shoots was calculated as described in the Section 3. Values represent the means ± SD of 12–30 samples. Statistical analysis using the Kruskal–Wallis test revealed no significant differences among ecotypes.

**Figure 4:**
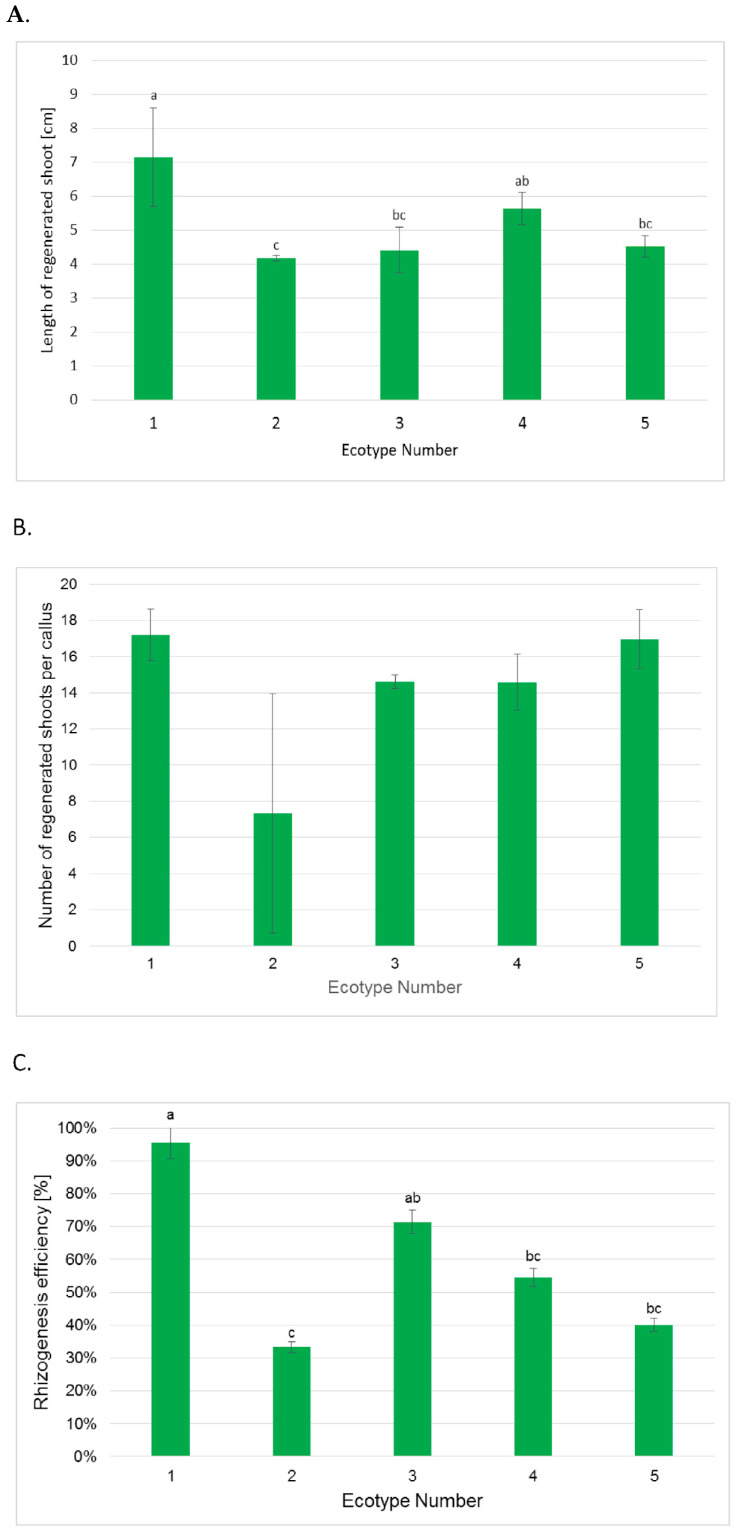
Length of regenerated shoots (**A**), number of regenerated shoots per tested callus (**B**), and efficiency of rhizogenesis (**C**) evaluated for five ecotypes of the Silesia cultivar, as described in the Section 3. Values are presented as means ± SD. One-way ANOVA followed by the least significant difference (LSD) test was used for statistical analysis of (**A**,**C**); different letters in these graphs indicate statistically significant differences at *p* ≤ 0.05. For (**B**), differences among ecotypes were assessed using the Kruskal–Wallis test; no significant differences were found (*p* > 0.05).

Although the callus frequency reached 100% for all analyzed ecotypes, the formed calli did not exhibit such high shoot regeneration (Figure 3). This parameter varied for different ecotypes; the highest was observed for ecotypes 1, 4 and 5; for these calli, all explants regenerated shoots. In the case of ecotype 2, a two-fold lower value was established for efficiency of shoot regeneration. For ecotype 3, 30% lower efficiency of shoot regeneration was observed when compared to the most effective ecotypes. The calculated correlation coefficient for the ratio of linoleic to α-linolenic acid in flax seeds and shoot regeneration efficiency was −0.78, which indicates a strong negative correlation. Thus, the higher the ratio of C18:2 to C18:3 fatty acids in seeds was, the lower was the ability of explants derived from these seeds to regenerate. The plants from ecotypes 1, 4, and 5 also demonstrated the greatest length of regenerated shoots (Figure 4A). The best results were observed for ecotype 1, for which almost two-fold longer regenerants were observed than for ecotype 2, which also exhibited an almost 50% lower efficiency of shoot regeneration than ecotype 1. For each callus, the number of regenerants was calculated (Figure 4B). It should be pointed out that the Silesia cultivar is characterized by very high regeneration efficiency: for ecotypes 1 and 5, as many as 17 shoots were obtained from one callus, and the developing shoots did not show vitrification. For ecotypes 3 and 4, the applied protocol allowed about 14 shoots per explant to be achieved. Ecotype 2, with the lowest regenerative efficiency, regenerated about seven shoots for each used explant. Thus, the obtained results are very promising, and for the other flax cultivar, such results were never obtained. It has been reported that flax explants (hypocotyl segments) usually produce 5 to 10 shoots [7,24]. Thus, it can be assumed that the observed yield of shoot regeneration for the Silesia cultivar of all ecotypes is significantly higher, which indicates a high morphogenetic potential of the tested cultivar.

The obtained shoots were cut and transferred to a basal medium used for flax growth. After 4 weeks, the rhizogenesis efficiency of the analyzed plants was measured, and the highest value of this parameter was again confirmed for ecotype 1 (Figure 4C).

Thus, we can assume that a certain relationship exists between the regenerative potential and the linoleic to α-linolenic acid ratio. Ecotypes (1 and 5) with an equivalent ratio of both unsaturated fatty acids are characterized by the highest regenerative potential. However, the other ecotypes do not follow any simple relationship related to the ratio of both unsaturated fatty acids, which suggests that in addition to the level and ratio of linoleic to α-linolenic acid, it would be beneficial to consider in the future the participation of the products of metabolism and autoxidation of both acids in the process of callogenesis and organogenesis. Therefore, although the influence of seed fatty acids on these processes is unquestionable, the nature of this influence remains unknown. So far, there are few reports on the relationship between polyunsaturated fatty acids and callogenesis and organogenesis. There is some limited evidence suggesting that fatty acids play a key role in restricting the ability of the pericycle to form a callus and thus the regenerative capacity of plants, including *Arabidopsis* [9].

### 2.4. Chlorophyll Measurements in Regenerated Plants

**Figure 5:**
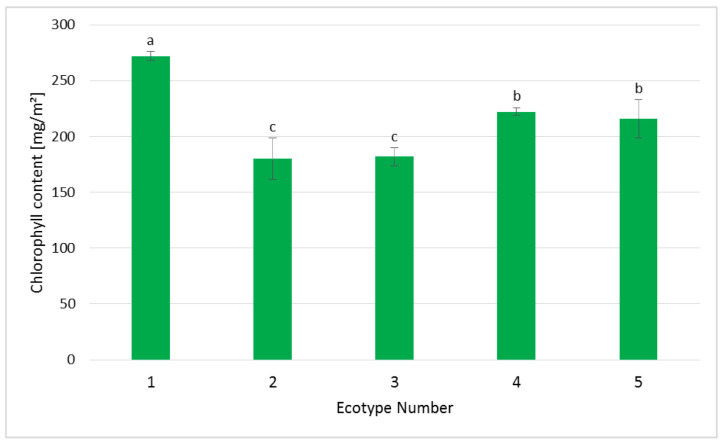
Chlorophyll content measured in regenerants of five ecotypes of the Silesia cultivar. The pigment was measured via the fluorescence ratio technique, as described in the Section 3. Values are presented as means ± SD of 5–8 samples. One-way ANOVA followed by the least significant difference (LSD) test was used for statistical analysis. Different letters indicate statistically significant differences at *p* ≤ 0.05 within each column.

In regenerated plants derived from different ecotypes of Silesia, the chlorophyll level was analyzed according to the method described in the Section 3. The obtained data indicate that the highest amount of chlorophyll was measured again in ecotypes 1, 4, and 5 (Figure 5). The same ecotypes also showed the highest regenerative properties and the highest length of regenerated shoots. Chlorophyll is a pigment crucial for photosynthesis and is a parameter which allows the physiological status of plants to be monitored [25]. The obtained data confirm this statement. Thus, the regenerants of ecotypes 1, 4, and 5 with the highest chlorophyll content were characterized by the most effective shoot regeneration. While plants from ecotype 2 possessed the lowest chlorophyll level, they were also derived from the callus with the lowest ability to regenerate.

The amount of chlorophyll reflects the physiological status of the plant [25] and potentially efficient regeneration in plant tissue culture. It should be emphasized that a relationship between the regenerative potential of the callus and chlorophyll content in regenerants was observed, since the ecotypes with the highest efficiency of shoot regeneration from the callus, i.e., 1, 4, and 5 (Figure 3), also exhibited the highest level of chlorophyll in regenerated shoots (Figure 5). It should be pointed out that chlorophyll content also reflects the rhizogenesis efficiency, and the calculated correlation coefficient for these two parameters was 0.66. The observed relationships are in accordance with those reported previously for other plants, e.g., pelargoniums [26].

### 2.5. Polyphenol and Flavonoid Content in Regenerated Plants

**Figure 6:**
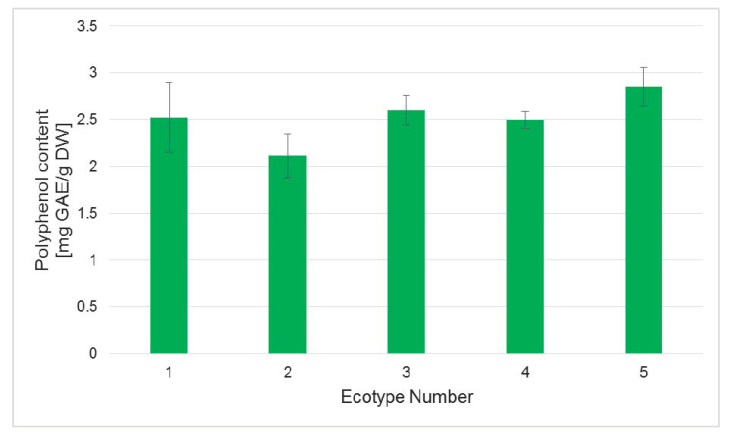
Content of polyphenols measured in regenerants of five ecotypes of flax Silesia cultivar. Analyses were performed in plants derived from tissue cultures, as described in the Section 3. Values represent the means ± SD of three samples. One-way ANOVA followed by the least significant difference (LSD) test was used for statistical analysis; no significant differences among ecotypes were found (*p* > 0.05).

The measurements of polyphenol and flavonoid content in the obtained regenerants of different Silesia ecotypes confirmed the data obtained in the regeneration of tissue cultures. Thus, the regenerants from ecotypes 1 and 5 had the highest amounts of measured polyphenols (Figure 6). The lowest level of these metabolites was noted for plants from ecotype 2, and even the observed changes were not statistically important; they were in agreement with other measured parameters, i.e., the efficiency of regeneration and chlorophyll level, which were the lowest for plants from ecotype 2.

**Figure 7:**
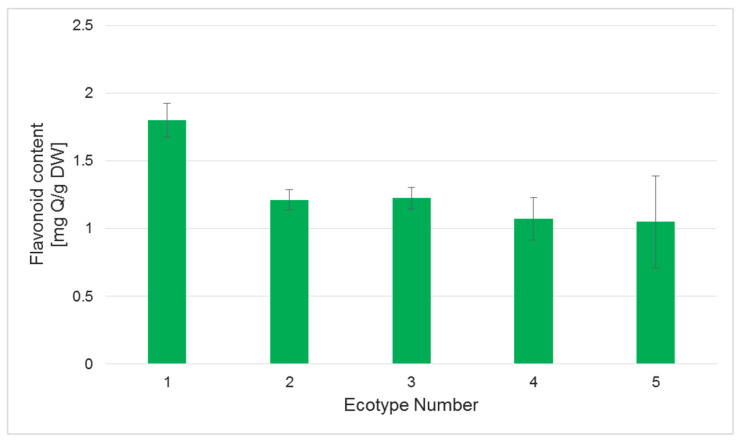
Levels of flavonoids in regenerants of five tested ecotypes of the Silesia cultivar. Measurements were performed as described in the Section 3. Values represent the means ± SD of three samples. One-way ANOVA followed by the least significant difference (LSD) test was used for statistical analysis; no significant differences among ecotypes were found (*p* > 0.05).

Different results for the level of flavonoids were observed. A comparable level of flavonoids was measured for the regenerants of ecotypes 2–4, while plants of ecotype 1 had the highest measured amount of flavonoids (Figure 7). These results are very interesting, especially as flavonoids are also treated as metabolites which influence developmental processes in plants [27,28]. Thus, it was noted that flavonoids may regulate the proteins responsible for the growth of plant cells and can affect the activity of IAA-oxidase, so they influence the level of plant growth regulators [28]. Indole-3-acetic acid (IAA) is the main auxin in plants, and it acts in plant developmental processes by influencing plant cell division, differentiation, and growth [29]. In fact, there is a relationship between flavonoid level and the length of regenerated shoots (Figure 4A); the calculated correlation coefficient between these two parameters was 0.79, which indicates a strong relationship and points to the role of flavonoids in regulation of plant growth.

## 3. Materials and Methods

### 3.1. Plant Material

All the flax seeds used in this study were provided by Professor Jan Szopa and were derived from cultivation in 2023 in the experimental field of Wroclaw University of Environmental and Life Sciences. First the seeds of Silesia cultivar were harvested from the individual plants, grown in different locations, and then they were propagated for 4 years in the field cultivation. Harvested seeds were selected by determining the fatty acid composition, which was a stable feature giving 5 ecotypes. Thus, the flax ecotypes with unique fatty acid profiles were obtained, and they are the research objects of the present study.

### 3.2. Establishment of Flax Tissue Cultures

The seeds of the Silesia cultivar were sterilized in a solution of 50% PPM (Plant Preservative Mixture) and then germinated on MS medium [30] supplemented with 1% sucrose and 0.8% agar (pH 5.8) for about 14 days in the darkness in a phytotron. After that time, the obtained young seedlings were exposed to the following photoperiod: 16/8 h (light/dark) at 21 °C/16 °C at 60% humidity.

### 3.3. Establishment of Germination Efficiency

In order to determine the germination efficiency of the analyzed ecotypes of Silesia seeds, the amounts of germinated seeds were counted per total number of seeds and expressed as a percentage.

### 3.4. Induction of Callus

The callus was initiated as described by Wróbel-Kwiatkowska et al. [3]. Thus, young seedlings (only cotyledons) were used for explant formation, which were transferred to callus induction medium (CIM). This medium contained MS medium, 1 mg/L BAP, 0.05 mg/L NAA, 2.5% glucose, 2.5% sucrose, 0.8% agar (pH 5.8). The explants were observed and transferred to fresh CIM medium every 14 days until callus formation. The regenerated calli were counted according to the following equation:

CF (calli frequency) = number of formed calli/number of cotyledon explants × 100%

### 3.5. Regenerations of Shoots

In order to regenerate shoots on obtained calli, shoot induction medium (SIM) was applied. This medium contained MS medium, 0.02 mg/L BAP, 0.001 mg/L NAA, 2.5% sucrose, and 0.8% agar (pH 5.8). Generated calli were cultivated on SIM medium in a phytotron (under the described conditions), until the shoots were formed. To induce root proliferation, regenerated shoots were cut and transferred to basal medium for flax growth (MS, 1% sucrose, 0.8% agar) without supplementation of any plant growth regulators. The regeneration capacity was established according to the following equation:

RS (efficiency of shoot regeneration) = number of regenerated shoots/number of callus explants × 100%

### 3.6. In Vitro Rooting of Obtained Regenerants

The plants regenerated from calli of 5 ecotypes of the Silesia cultivar were cut and rooted in basal medium for flax cultivation (MS supplemented with 1% sucrose and 0.8% agar). The rooting efficiency was assessed according to a 5-step scale and calculated according to the following equation:

RE (rooting efficiency) = Ʃ(v × n)/(V × N) × 100%

where

v—level of rooting assessed according to 5-step scale;
n—number of shoots at each assessed level;
V—the highest level of the used scale;
N—total number of shoots taken for rooting.

### 3.7. Determination of Fatty Acid Profile in Silesia Seeds

Determination of levels of fatty acids in selected seeds of the Silesia cultivar was performed using gas chromatography with flame ionization detection (GC-FID).

Briefly, 100 mg of homogenized seeds was extracted with hexane in a solvent-to-sample ratio of 30:1 (*v*/*w*). The mixture was shaken for 15 min at 30 °C, centrifuged, and the supernatant collected. The extraction step was repeated, and the combined supernatants were concentrated to a final volume of 1 mL. An aliquot of 125 μL was transferred into a 15 mL DURAN glass tube, evaporated to dryness, and subjected to transmethylation according to Hasiewicz-Derkacz et al. [31].

The resulting fatty acid methyl esters (FAMEs) were analyzed by gas chromatography. Separations were carried out on an SP™-2560 fused silica capillary column (Supelco, Bellefonte, PA, USA; 100 m × 0.25 mm i.d., 0.20 μm film thickness) using an Agilent 6890N GC system equipped with a flame ionization detector (Agilent Technologies, Santa Clara, CA, USA). Detailed chromatographic conditions and the temperature program were described previously [32]. Fatty acids were identified by comparing the retention times of peaks with those of a reference FAME mixture (Supelco 37 Component FAME Mix, Supelco, Bellefonte, PA, USA). Fatty acid composition was expressed as the relative percentage of each fatty acid in the total fatty acid pool.

### 3.8. Chlorophyll Level in Regenerated Plants

The chlorophyll content was determined using an OptiSciences CCM300 Chlorophyll Content Meter (OptiSciences, Hudson, NH, USA) by measuring the chlorophyll fluorescence emission ratio of intensity at 735 nm/700 nm after excitation with 460 nm blue LED with a half bandwidth of 15 nm, according to the reference method described by Gitelson et al. [33]. The measurements were made on the upper leaf epidermis in triplicate, with samples taken from three different internodes using a fiber optic cable with a clip attached to the instrument. Chlorophyll content is presented in mg/m^−2^.

### 3.9. Total Polyphenol and Flavonoid Content in Regenerated Plants

Total polyphenol and flavonoid contents were extracted with ethanol from lyophilized tissue of Silesia regenerants as described by Liszka et al. [34]. Thus, 70% ethanol was used for extraction of lyophilized tissue of regenerated plants, and obtained extracts were incubated in an ultrasonic bath at 60 °C for 45 min, followed by centrifugation.

In order to measure the amounts of polyphenols, a spectrophotometric method was applied, as was a colorimetric method for flavonoid content measurements.

In the aim to determine polyphenols, 250 µL of the ethanol extract was transferred to glass test tubes, followed by the addition of 1.25 mL of 10-fold diluted Folin–Ciocalteu reagent. After incubation in the dark at room temperature for 5 min, 1 mL of Na_2_CO_3_ solution (75 g/L) was added. Samples were then incubated at room temperature for 30 min and used for absorbance at 750 nm. All absorbance measurements were performed using a Tecan microplate reader (Tecan Group Ltd., Männedorf, Switzerland). The obtained results were expressed as milligrams of gallic acid equivalents (GAE eq.) per gram of dry weight (lyophilized tissue).

Total flavonoids content was determined in the ethanol extract performed as described above. An amount of 1 mL of the supernatant was mixed with 300 µL of 5% NaNO_2_ and incubated for 5 min at room temperature. Subsequently, 500 µL of 2% AlCl_3_ was added, and the mixture was incubated for an additional 5 min. Finally, 500 µL of 1 M NaOH was added, the samples were mixed, incubated for 10 min, and the absorbance was measured at 510 nm using a Tecan microplate reader as described above. The flavonoid level was expressed as quercetin equivalents (QE eq.) and calculated per gram of plant dry weight.

### 3.10. Statistical Analysis

Statistical analysis was performed using Statistica 14 software (TIBCO Software Inc., Palo Alto, CA, USA) by applying both parametric and non-parametric statistical methods. One-way analysis of variance (ANOVA) was employed to evaluate differences among treatment groups, with post hoc comparisons conducted using the least significant difference (LSD) test at a significance level of *p* < 0.05. In cases where data violated the assumptions of parametric testing, the Kruskal–Wallis test was utilized, followed by Dunn’s multiple comparison test with Bonferroni correction. A threshold of *p* < 0.05 was considered statistically significant for all analyses.

## 4. Conclusions

These findings support the very high regenerative potential of the new Polish flax Silesia cultivar. It should be pointed out that regenerative capacity was correlated with chlorophyll content and polyphenol amount. Ecotype 1 was characterized by the best regenerative properties and the highest measured level of the analyzed metabolites—chlorophyll and flavonoids—and belonged to the group with the highest polyphenol levels. Similar observations of high regeneration frequency and a very large number of regenerated plants assessed per callus were made for ecotype 5. For regenerants of this ecotype, improved regenerative properties were accompanied by the largest amounts of polyphenols and increased levels of chlorophyll in comparison to the other ecotypes (ecotype 2 and 3). It can be assumed that for these two ecotypes (1 and 5), a similar biochemical composition of fatty acids in the seeds was determined. Thus, the levels of all measured fatty acids were similar; the linoleic to α-linolenic acid ratio was 1.36 for seeds of ecotype 1 and 1.68 for seeds of ecotype 5. The amounts of the other fatty acids (palmitic, stearic, and oleic acids) were comparable in the tested seeds of both ecotypes (1 and 5).

The other analyzed ecotypes exhibited a ratio of C18:2:C18:3 equal to 55.28 (ecotype 2), 0.36 (ecotype 3), and 6.23 (ecotype 4). Thus, the calculated linoleic to α-linolenic acid ratio in the seeds of these ecotypes was far from that observed for ecotypes 1 and 5.

The amounts of the other fatty acids in ecotypes 2–4 were at the same level, except for seeds from ecotype 3, with a higher level of oleic acid.

These data suggest that, among many factors which influence the regenerative potential of plants, there is a potential role of the fatty acid composition in the seeds from which explants for regeneration are obtained. In fact, the only identified factor differentiating the explants was the composition of polyunsaturated fatty acids of the seeds; the other internal factors such as the type of explant, plant age, and genotype, as well as external factors such as the culture medium, temperature, and light, were identical. Despite this, quantitative differences in callus formation and regeneration were observed. The obtained data suggest a previously unidentified role of the PUFA ratio in determining callus formation capacity and its regenerative potential, possibly through polyunsaturated-fatty-acid-mediated signaling pathways in plant metabolism. It should be also pointed out that the only analyzed parameter in the present study was fatty acid amounts and ratio, but in fact many other parameters/compounds could influence callus formation, shoot regeneration, and root forming. In fact, they were not analyzed in this research. In the near future, the level of genome methylation of individual ecotypes will be investigated.

The other important result is the determination of the very high morphogenetic capacity of a flax cultivar (Silesia) for which no analyses have been performed in tissue cultures so far. The observed parameters, especially those concerning callus formation and the frequency of shoots, indicate that the characterized flax cultivar (Silesia) may be applied with high efficiency for regeneration in tissue cultures and micropropagation. The reason for this might be the genotype of the tested cultivar and the protocol applied for callus formation and organogenesis.

In the near future, the described Silesia cultivar, characterized by optimal regenerative properties, will be applied for genetic engineering to obtain plants with novel features.

## Figures and Tables

**Table 1:** Fatty acid composition in the seeds of five tested ecotypes of the Silesia flax. The measurements were performed by the GC-FID method as described in the Section 3 and expressed as the relative percentage of each fatty acid in the total fatty acid pool. Values are presented as means ± SD of three samples. One-way ANOVA followed by the least significant difference (LSD) test was used for statistical analysis. Different letters indicate statistically significant differences at *p* ≤ 0.05 within each column.

Content of Individual Fatty Acids in Selected Ecotypes of the Silesia Flax					
Ecotype Number	Palmitic Acid (C16:0)	Stearic Acid (C18:0)	Oleic Acid (C18:1)	Linoleic Acid (C18:2)	α-Linolenic Acid (C18:3)
1	4.99 ± 0.09 a	5.93 ± 0.32 a	15.82 ± 0.05 b	42.29 ± 0.87 c	30.96 ± 0.52 b
2	4.78 ± 0.39 a	3.39 ± 0.11 b	11.91 ± 1.18 c	78.50 ± 1.58 a	1.42 ± 0.13 e
3	4.47 ± 0.14 a	6.47 ± 0.31 a	25.44 ± 0.43 a	16.96 ± 0.44 d	46.66 ± 0.15 a
4	5.21 ± 1.04 a	6.76 ± 0.52 a	14.44 ± 2.12 bc	63.43 ± 0.58 b	10.17 ± 0.03 d
5	4.68 ± 0.46 a	7.28 ± 1.633 a	17.51 ± 1.03 b	44.23 ± 1.25 c	26.29 ± 1.11 c

**Table 2:** The calculated ratio of the content of linoleic (C18:2) to α-linolenic (C18:3) fatty acids for the seeds of 5 tested ecotypes of Silesia cultivar.

Ecotype of Silesia	1	2	3	4	5
Ratio of linoleic to α-linolenic acid	1.36	55.28	0.36	6.23	1.68

## Data Availability

The original contributions presented in this study are included in the article. Further inquiries can be directed to the corresponding author.
